# Reversible Addition‐Fragmentation Chain‐Transfer Polymerization in Supercritical CO_2_: A Review

**DOI:** 10.1002/marc.202400514

**Published:** 2024-09-11

**Authors:** Friso G. Versteeg, Francesco Picchioni

**Affiliations:** ^1^ Department of Chemical Engineering – Product Technology University of Groningen Nijenborgh 4 Groningen 9747 AG The Netherlands

**Keywords:** controlled radical polymerization, supercritical CO_2_

## Abstract

The development of cleaner, more environmentally friendly processes in polymerization technology is crucial due to the prevalent use of volatile organic solvents (VOCs), which are harmful and toxic. Future regulations are likely to limit or ban VOCs. This review explores the use of supercritical solvents, specifically supercritical CO_2_ (scCO_2_), in polymerization processes. The study focuses on reversible addition‐fragmentation chain‐transfer (RAFT) induced homo‐polymerization of various monomers using specific chain transfer agents (CTAs) in scCO_2_. RAFT polymerization, a reversible deactivation radical polymerization (RDRP) polymerization, relies heavily on the choice of CTA, which significantly influences the dispersity and molar mass of the resulting polymers. Stabilizers are also crucial in controlling product specifications for polymerizations in supercritical CO_2_, except for fluor‐based polymers, although they must be removed and preferably recycled to ensure product purity and sustainability. The review notes that achieving high molar mass through RAFT polymerization in scCO_2_ is challenging due to solubility limits, which lead to polymer precipitation. Despite this, RAFT polymerization in scCO_2_ shows promise for sustainable, circular production of low molar mass polymers, although these cannot yet be fully considered green products.

## Graphical abstract

This section introduces RAFT polymerization, emphasizing its role in controlled polymer synthesis. It explores supercritical CO_2_ as a solvent, highlighting its properties, advantages, and environmental benefits. The RAFT polymerization mechanism in supercritical CO_2_ is detailed, focusing on initiation, propagation, and termination steps. Additionally, an overview of key literature on homopolymerization and block copolymerization is provided.

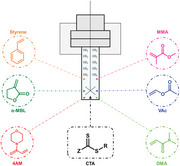

## 1 Introduction

The development of new processes that are cleaner and more environmentally friendly is one of the main challenges in polymerization.^[^ ^1^, ^2^ ^]^ Although a lot of attention has already been paid to the reduction of greenhouse gas emissions such as carbon dioxide, methane and NO_x_, there are more environmentally harmful chemicals that are causing also major issues.^[^ ^3^ ^]^ An example of a class of overlooked compounds in polymerization processes is the organic solvents comprising of, alcohols, ethyl ether, hexane, toluene and xylene.^[^ ^4^ ^]^ Each one is capable of dissolving other chemicals, especially nonpolar chemicals. To enable chemical reactions, it is usually necessary to dissolve the reactants in a solvent. A major drawback of those organic solvents is their volatility, consequently, these chemicals are normally referred to as volatile organic compounds (VOCs).^[^ ^5^ ^]^ Furthermore, these VOCs mostly originate from fossil sources which are another reason to explore sustainable for alternatives for the replacement of these solvents.^[^ ^4^ ^]^

Currently, there are new ways being investigated to replace the VOCs in polymer production processes. Possible options to stop using and replace organic solvents in, e.g., include water, biomass‐derived solvents, ionic liquids and supercritical fluids.^[^ ^6^ ^]^ The advantages of using water for organic synthesis are that it is safe to handle, non‐toxic and environmentally friendly.^[^ ^7^, ^8^ ^]^ Sometimes the change to another solvent also has other beneficial advantages such as a reduction in byproduct formation or the synthesis can be performed under milder reaction conditions. This was shown in the acceleration on the Diels–Alders reaction by Breslow et al.^[^ ^9^ ^]^ It was stated that the solubility of the substrate/reactants in water is not always linearly proportional to the reaction rate.^[^ ^10^, ^11^ ^]^ Sharpless et al. reported a remarkable enhancement in the observed reaction rate of organic compounds dissolved in an aqueous suspension.^[^ ^12^ ^]^ The reactions in this system occur at the interface of the water and organic compounds, with the latter being the reactant phase; these reaction mechanisms are also being driven by the important presence of a catalyst. Both organic and inorganic catalysts are gaining increasingly more as a mean to help replace organic solvents with aqueous solutions in industry, therefore reducing the environmentally unfriendly waste stream that are obtained as undesirable by‐products.^[^ ^13^ ^]^ There is already a vast amount of organic reactions occurring with the help of a water‐compatible catalyst carried out in aqueous solutions. For instance, Kitanosono et al. published a review of nonmetal, metal‐based and surfactant‐based catalytic reactions in aqueous solutions.^[^ ^14^ ^]^

Similar to the use of water as a solvent, ionic liquids seem to be attractive solvent alternatives to conventionally used VOCs. Ionic liquids are defined as salts that are present in their liquid state at room temperatures or temperatures below 100 °C.^[^ ^15^ ^]^ The advantages of using ionic liquids instead of organic solvents are low vapor pressure under ambient temperature and pressure thus making most of them nonflammable.^[^ ^16^ ^]^ Nevertheless, the high cost associated with ionic liquids is a major drawback that prevents them from becoming readily useable. It is believed that the prices are between 5 and 20 times higher than organic solvents.^[^ ^17^ ^]^ This does not mean that there was not any research performed to the functionality of ionic liquids. Qureshi et al. has published an extensive review of the applications of ionic liquids in organic chemistry, catalysis chemistry, polymer chemistry, etc.^[^ ^15^ ^]^

An alternative to the options described above is provided by supercritical fluids. A supercritical fluid was discovered for the first time in 1820 by Cagniard de la Tour.^[^ ^18^ ^]^ Cagniard filled a Papin's digester with a liquid in which a flint ball was placed. By heating this system far above the boiling point of the liquid in a closed vessel, it was noted that the splashing sound of the flint ball in the digester was reduced substantially. From this it was concluded that there was no liquid–gas phase boundary anymore and therefore no surface tension. This newly observed fluid was called a supercritical fluid, in which the density is similar to that of a liquid but the diffusivity resembles more to a gas.^[^ ^18^ ^]^ Over the past two to three decades there has been an increasing interest in the application of supercritical fluids.

## 2 Supercritical CO_2_

**Figure 1:**
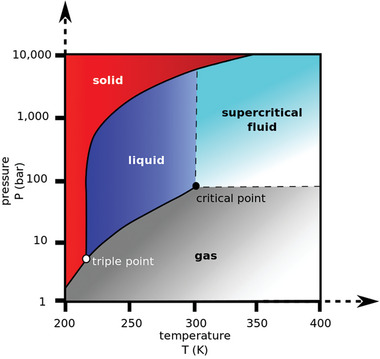
Phase diagram of carbon dioxide.

One of the most researched supercritical fluids is supercritical CO_2_. A substance can be classified as supercritical when it is above its critical parameters (namely pressure and temperature), as seen in **Figure** **1**. Supercritical CO_2_ stands out from other supercritical fluids due to its low critical point of only 304 K and 7.4 MPa.^[^ ^19^ ^]^ Not only are the critical parameters “easily” reached, but it is also nontoxic, non‐flammable and abundant. Due to CO_2_ being a waste product in many industries it can be repurposed in different processes, thus giving it a second life to delay greenhouse emissions.^[^ ^20^ ^]^

There are already implementations of scCO_2_ in industrial scale processes. One of the biggest processes where supercritical fluids are applied, is the extraction of compounds that are present in materials originating from biomass, which is usually referred to as supercritical fluid extraction. Most notably, the decaffeination from coffee with supercritical CO_2_ has performed industrially since the early 1970s, based on the patents of Zosel.^[^ ^21^ ^]^ Before the introduction of the supercritical extraction technique the decaffeination of coffee was done by using organic solvents, with methylene chloride being the best solvent for this process. Even though the remaining concentration of the organic solvent in the coffee beans was below 10 ppm, this still might have a negative impact on the overall health let alone the economic costs of drying.^[^ ^22^ ^]^

It is important to understand the physical properties of scCO_2_ before a process can be built around it. Although CO_2_ itself is an inert gas at room temperature and pressure, meaning that it will not participate in reactions, there are a few limitations.^[^ ^23^, ^24^ ^]^ From a chemical structure point of view, it can clearly be seen that scCO_2_ is a very non‐polar solvent with a low dielectric constant, and no dipolar moment.^[^ ^24^, ^25^ ^]^ This would also imply that in theory the solubility of organic, polar molecules would be rather limited. This means that other non‐polar compounds like benzene, pentane and hexane show good affinity toward scCO_2_. Nonetheless, the quadruple moment of scCO_2_ suggest that low polar chemicals should exhibit reasonable to some solubility in scCO_2_, even though this hinders the affinity toward non‐polar compounds.^[^ ^26^, ^27^ ^]^ Furthermore, it was stated that on a molecular level CO_2_ can exhibit both functions as a weak Lewis base as well as a weak Lewis acid.^[^ ^28^, ^29^, ^30^ ^]^ Finally, it is suggested that CO_2_ can participate in conventional and nonconventional hydrogen bonding.^[^ ^24^ ^]^ Raveendran et al. has published an in depth study about the polar attributes of supercritical CO_2_ and how it compares to polar solvents like, e.g., water.^[^ ^24^ ^]^

These properties, in combination with the abundance of CO_2_ and its non‐toxicity, make it a great source for a variety of applications. Besides the more common usage as an extraction solvent it is also possible to use it for conventional solvent chemistry.^[^ ^31^ ^]^ It can replace the organic solvent in extraction chemistry but it can also be used as a solvent for synthesis chemistry.^[^ ^32^, ^33^ ^]^ In recent years supercritical CO_2_ gotten attention as a medium for polymer chemistry.^[^ ^34^ ^]^ Most of the non‐polar organic monomers are soluble in supercritical CO_2_, at the right temperature and pressure. This point when the monomer becomes soluble in scCO_2_ is called the cloud point and thus below the cloud point the monomer is not soluble in CO_2_.^[^ ^35^, ^36^ ^]^ The benefit of a CO_2_ medium is that in general the purification of the polymer is relatively easy. By releasing the pressure, the unreacted monomer and CO_2_ are leaving the reactor and the pure polymer is left behind. This has also attracted the interest of performing controlled radical polymerization (CRP) in supercritical CO_2_, with one of them being reversible addition‐fragmentation chain transfer polymerization (RAFT).

## 3 RAFT Polymerization

**Figure 2:**
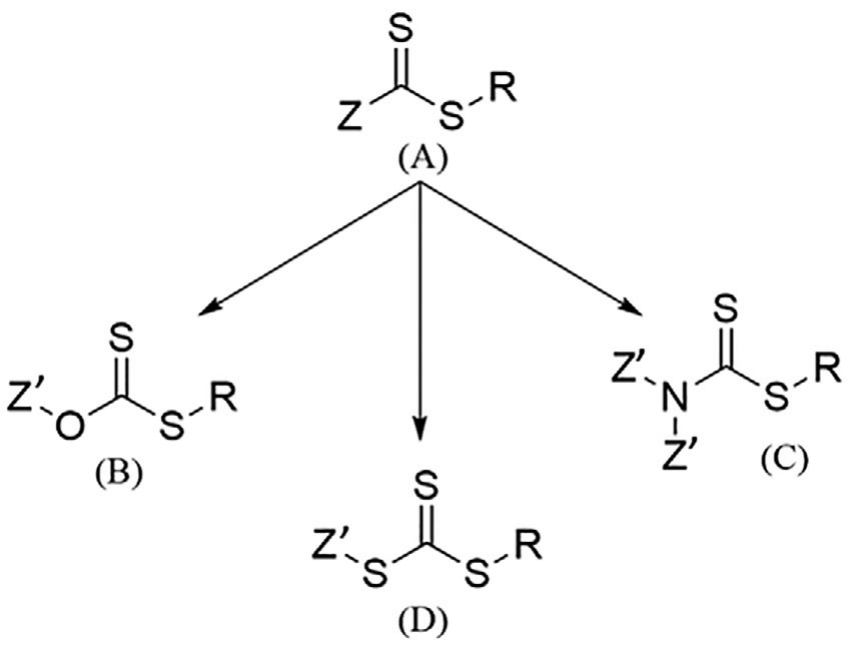
A) General chemical structure of CTAs with Z being the stabilizing group and R the leaving group, with three typical CTA structures; B) Xanthate, C) Dithiocarbamates, D) Trithiocarbonates.

In recent years reversible deactivation radical polymerization, has received high attention. This concept is different from the already known free radical polymerization. One of these forms is called reversible addition‐fragmentation chain transfer (RAFT) polymerization, developed in 1998.^[^ ^37^ ^]^ Two other well‐known techniques are nitroxide‐mediated radical polymerization (NMP) and atom transfer radical polymerization (ATRP). The benefits of these reversible deactivation radical polymerization (RDRP) are the narrow molar mass distribution and well‐defined particle structures.^[^ ^37^, ^38^ ^]^ RAFT polymerization has some advantages over ARTP and NMP such as the effectiveness over a wide range of temperatures, high tolerance in the functionality in monomer and solvent and the wide range of reaction conditions.^[^ ^39^, ^40^ ^]^ The key to a good RAFT polymerization is the right choice of chain transfer agent (CTA). The CTA consists either of dithio‐ or trithio molecules. For example, dithioester, dithiocarbamates and trithiocarbonates and xanthates are molecules that are frequently being used (**Figure** **2**).^[^ ^41^, ^42^, ^43^, ^44^ ^]^ Furthermore, it includes a Z group that stabilizes and regulates the activation of the C═S bond, along with an R group that serves as the leaving group. For methacrylate polymerizations dithiobenzoates and trithiocarbonates are often used.

**Figure 3:**
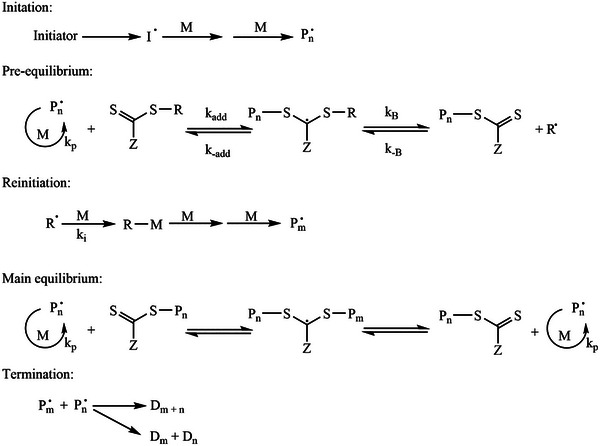
Schematic drawing of the RAFT mechanism.

The process, depicted in **Figure** **3**, starts with the decomposition of an initiator into a radical which will react with a monomer to form a propagating radical i). After the initial propagation the polymeric radical reacts with the CTA. The selection of the a suitable CTA is crucial for the effectiveness of the polymerization. A fragmentation reaction may occur to form a new radical and a polymeric CTA (pre‐equilibrium) ii). The newly formed radical will undergo re‐initiation with a monomer that will consecutively start a new polymeric chain. A more detailed explanation of which CTA(s) to choose for which systems are given in other reviews, in these studies the selection of the R and Z groups is also thoroughly discussed.^[^ ^40^, ^45^ ^]^ In the main equilibrium iii) polymeric growth is occurring between the radical species that were not yet subjected to a termination reaction. The last step iv) is the termination reaction where the growth of the polymeric chain is stopped and the remaining polymer molecule becomes a “dead polymer chain”. The term “dead polymer chain” refers to a polymer chain that has lost its ability to participate in further polymerization reactions. This loss occurs because the active radical site at the end of the chain has been terminated in a way that it can no longer react with monomers or other radicals.

**Figure 4:**
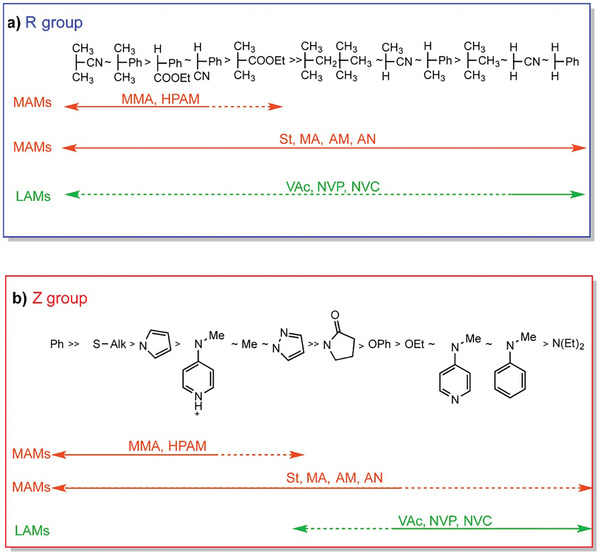
Compatible R/Z‐groups for MAMs and LAMs taken from Perrier and Keddie et al. with copyright permission from Macromolecules.^[^ ^40^, ^46^ ^]^ a) Transfer coefficients decrease from left to right. Fragmentation rates also decrease from left to right. A dashed line indicates partial control (i.e., control of molar mass but poor control over dispersity or substantial retardation in the case of VAc, NVC, or NVP). b) Addition rates decrease and fragmentation rates increase from left to right. A dashed line indicates partial control (i.e., control of molar mass but poor control over dispersity or substantial retardation in the case of LAMs such as VAc or NVP).

A suitable CTA for the RAFT polymerization depends mainly on the monomer and initiator used. Vinyl monomers can roughly be divided into two groups, “more activated” monomers and “less activated” monomers. “More activated monomers” (MAM) have a double bond conjugated to another double bond, an aromatic ring, carbonyl group or a nitrile while “less activate monomers” (LAM) exhibit a double bond adjacent to an oxygen, nitrogen, halogen, sulfur lone pair or saturated carbons.^[^ ^40^ ^]^ For a successful RAFT process, it must be ensured that the C═S bond is more reactive to radical addition than the C═C bond of the monomer. This is achieved by carefully selecting the Z group which is responsible for the reactivity of the C═S bond toward radical addition and the stability of the intermediate. The R group affects the RAFT process in three ways, 1) radical addition to CTA, 2) subsequent fragmentation from the intermediate formed and 3) propagation. A fine balance exists between radical stability and steric effects, namely that the R‐group has to form a radical stable enough to be formed, and reactive enough so that it can add to a monomer. Perrier gave a nice overview of which Z and R group are most suited for conventional RAFT polymerization when using more activated monomers and less activated monomers as shown in **Figure** **4**.^[^ ^40^ ^]^

One of the major advantages of the RAFT polymerization is that the molar mass can be pre‐determined by the concentration of the monomer, CTA, conversion and in certain cases initiator concentration. The most commonly used formula to predict the molar mass of the final product is:

(1) $$M_{n,th}=\frac{{\left[M\right]}_{0}}{{\left[RA\right]}_{0}}\;*M_{W,m}*X+M_{W,Raft}$$

where M_n,th_ is the theoretical molar mass, [M]_0_ is the initial monomer concentration, [RA]_0_ is the initial CTA concentration, M_W,m_ is the molar mass of the monomer, X is the conversion and M_W,Raft_ is the molar mass of the CTA. In conventional solvents, RAFT polymerizations are typically carried out using a low amount of initiator (in comparison to CTA), for example RAFT:AIBN ratio of 10:1. When using scCO_2_ as solvent media a higher ratio of AIBN is typically used, RAFT:AIBN (1:1) or (2:1). If the concentration of AIBN is increased, albeit in organic or scCO_2_ solvent, it is important to take the decomposition of AIBN into consideration when estimating the theoretical molar mass. An addition to (1) gives a new equation for the theoretical molar mass.

(2) $$M_{n,th}=\frac{{\left[M\right]}_{0}}{{\left[RA\right]}_{0}+df\left({\left[I\right]}_{0}-{\left[I\right]}_{t}\right)}\;*M_{W,m}*X+M_{W,Raft}$$

where [I]_0_ is the initial initiator concentration, [I]*_t_* is the initiator concentration at time *t*. The initiator concentration at time *t*, in batch, is calculated by using the following relation:

(3) $${\left[I\right]}_{0}-\;{\left[I\right]}_{t}={\left[I\right]}_{0}\;\left(1-e^{-k_{d}t}\right)$$

where k_d_ is the decomposition rate constant of AIBN. However, when the RAFT concentration is much larger than the initiator concentrations, Equation (2) will be almost equal to Equation (1). A RAFT polymerization exhibits good control when the final product has i) a narrow dispersity (Đ) and ii) close resemblances between the molar mass and theoretical molar mass.

## 4 RAFT Dispersion Homopolymerization in Supercritical CO_2_

**Scheme 1:**
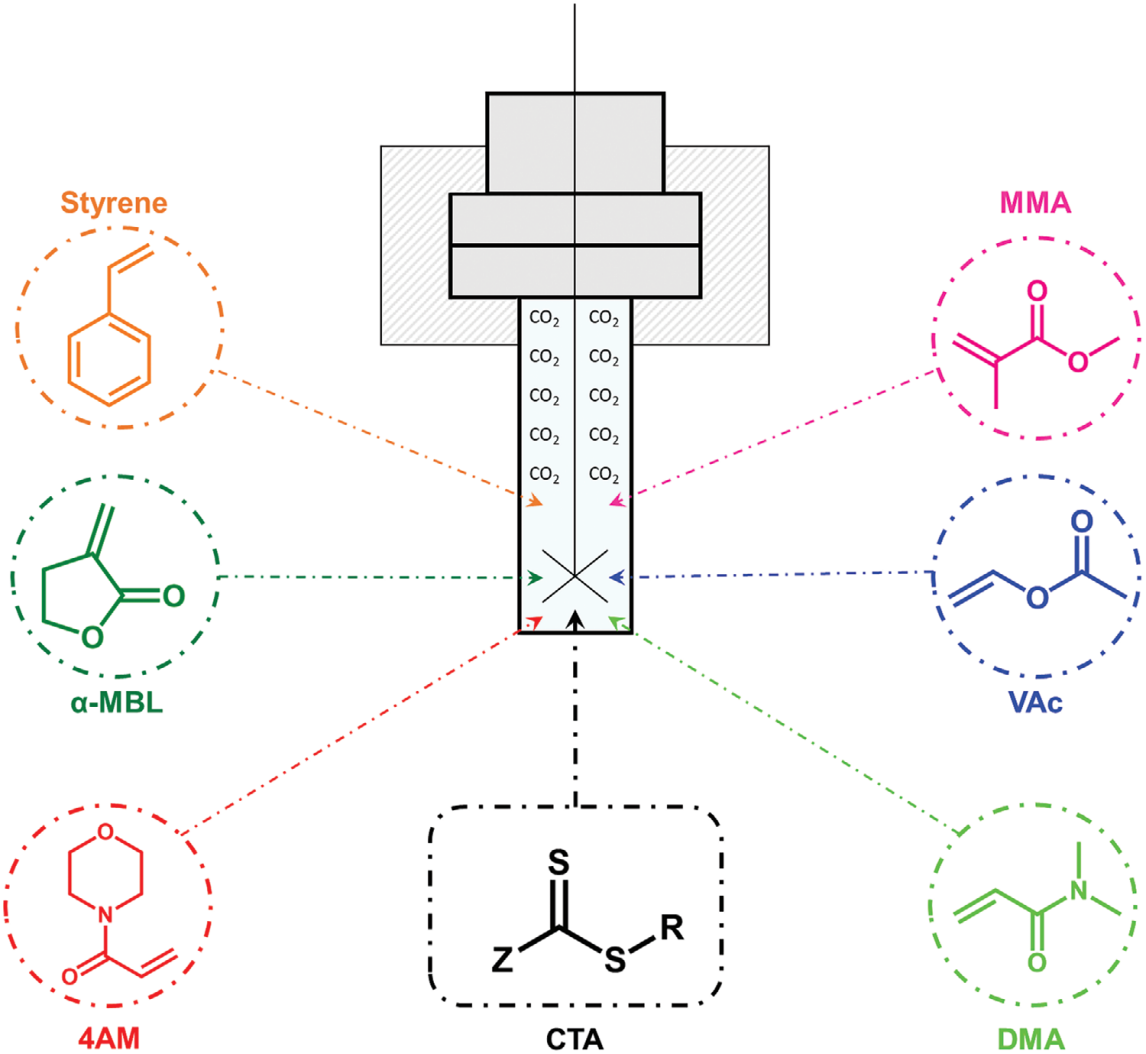
Monomers for RAFT polymerization in supercritical CO_2_ in a high pressure batch reactor.

The use of supercritical CO_2_ as a medium for RAFT mediated polymerization was first reported by Arita in 2004 where methyl methacrylate and styrene were used as monomers.^[^ ^47^, ^48^ ^]^ In the following years the RAFT polymerization in scCO_2_ was extensively researched and improved, **Scheme** **1**. More monomers and CTAs were developed and tested; the process is optimized by the incorporation of stabilizers. Desimone et al. did extensive research on the decomposition of AIBN in supercritical CO_2_ as it is a commonly used initiator in RAFT polymerization.^[^ ^49^ ^]^ It was observed that the decomposition rate (k_d_) of AIBN is significantly lower in scCO_2_ compared to organic solvents. For example, the k_d_ of AIBN is 1.6 times lower in supercritical CO_2_ compared to benzene. This is why in the published RAFT polymerizations in scCO_2_ the AIBN to RAFT concentration is noticeably higher. A concentration ratio of [1]:[1] is not uncommon.^[^ ^49^ ^]^

The precise mechanism of RAFT polymerization in scCO_2_ is still under debate, however, Pacheco et al. suggested a possible explanation.^[^ ^50^ ^]^ Mueller et al. modelled MMA radical polymerization in supercritical CO_2_.^[^ ^51^ ^]^ It was defined that the main locus of conventional radical polymerization are the polymeric microparticles. This means that at the beginning of the polymerization, microparticles are formed within the scCO_2_ phase. However when the critical length of the polymer is reached, it disperses out of the scCO_2_ phase. Then the main locus of the reaction becomes the polymeric particles in which new radicals that are generated in the scCO_2_ phase rapidly migrates to the dispersed phase before undergoing termination.

### 4.1 Stabilizers

In general according to Beckman and Rindfleisch a polymer is has good solubility in scCO_2_ when the polymer has i) a low *T_g_*, ii) a flexible backbone, iii) groups that have specific interactions with CO_2_.^[^ ^52^, ^53^ ^]^ Because not all polymers exhibit these properties, stabilizers have been added to the reaction mixture to ensure better solubility of the polymer and thus to assure that this polymer remains longer soluble in the homogenous phase. In other words, one of the roles of the stabilizer is to stabilize the nuclei formed during the initial stage of the reaction.^[^ ^50^ ^]^ This means that the polymer can continue to undergo propagation reactions and therefore grow larger before it disperses out of the solution. The stabilizer is also important to control the morphology of the final polymer particles. A suitable stabilizer needs to have a good solubility in scCO_2_. Three groups of stabilizers have been identified that show good affinity toward CO_2_; fluorinated, siloxane and hydrocarbon‐based stabilizers. The most commonly used stabilizers are the so‐called Krytox compounds (e.g., perfluoro‐ether) or molecules with siloxane attachment (e.g., PDMS‐MMA). The use of higher stabilizer concentrations leads to i) more spherical polymer particles (SEM), ii) a higher molar mass, iii) a higher polymer yield and iv) a lower dispersity index (Đ) of the product. It must be noted that an anchoring group must be present for successful stabilization for siloxane based stabilizer, which in most cases is methyl methacrylate. Furthermore, because PDMS‐MMA is a macromonomer, it is reported that up to 15% of the starting stabilizer concentration can be covalently bonded to the final product.^[^ ^54^, ^55^ ^]^ There has been no report on incorporation of fluorinated stabilizers in the polymer structure of the end product. However the use of fluorinated stabilizers can be regarded as unfavorable due to the potential negative effect on the environment if not recycled properly.^[^ ^56^ ^]^

### 4.2 CTA Selection for Supercritical CO_2_ Processes

**Figure 5:**
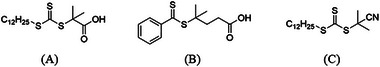
Chain transfer agent investigated by Pacheco et al. and redrawn; A) DDMAT; B) CPAB; C) CPDT.^[^ ^50^ ^]^

As discussed previously, the choice of the CTA is a key factor to a successful RAFT polymerization.^[^ ^57^ ^]^ However, in a recent study published by Pacheco et al., it showed that the suitability of the CTA differs from conventional solvents when supercritical CO_2_ is used.^[^ ^50^, ^58^ ^]^ For MMA, it was found that a CTA agent (DDMAT) which in conventional solution gives no control over the polymerization exhibited great control over the polymerization in scCO_2_. By this finding Pacheco tested the three CTAs for the polymerization of MMA, depicted in **Figure** **5**.

**Figure 6:**
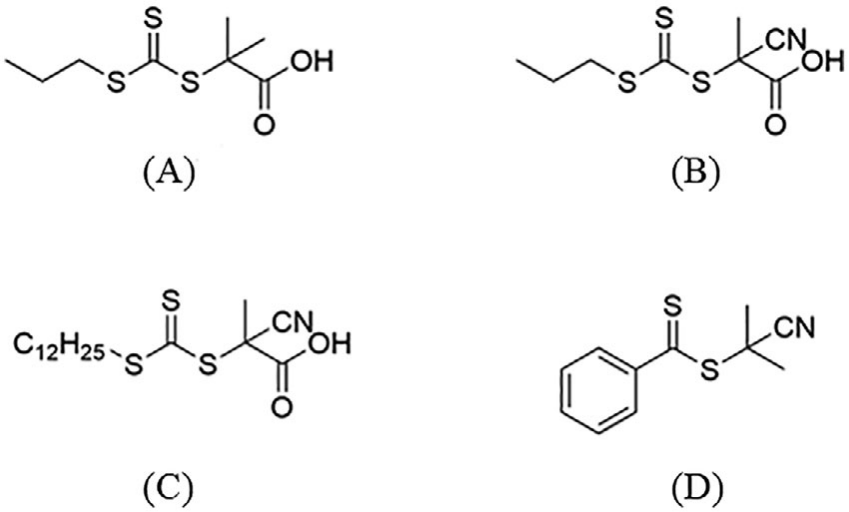
Additional CTAs investigated by Pacheco et al. and redrawn; A) PDMAT, B) CTPPA, C) CPAD, D) CPDB.^[^ ^50^ ^]^

From experimental results, DDMAT showed good control (low Đ)in scCO_2_ while its performance was poor in toluene. However, CPAB showed low control in scCO_2_ though the control was good in toluene whereas CPDT showed good control in both toluene and scCO_2_. To understand these trends, computational solvation models were used to simulate the affinities of the different CTAs toward scCO_2_ and toluene. It was concluded that unsaturated groups (C═O/C≡N) have a strong affinity with scCO_2_, whereas the C_12_H_25_ group from DDMAT and CPDT have not. Furthermore, the solubility of the CTAs plays a crucial role in the first step of the two‐step polymerization, as discussed in the previous section. For DDMAT this means that because of the generally low solubility it would quickly diffuse from the scCO_2_ phase to the particle phase which increases the DDMAT/MMA ratio. By having a high DDMAT/MMA ratio the low chain transfer constant of DDMAT will be countered. For CPAB the solubility in scCO_2_ is considerably causing lower diffusion of CPAB into the particle where the locus point of the reaction is. In this situation, there is a high MMA/CPAB ratio which results in loss of control in the reaction. To conclude this, a CTA must have (homogenous) solubility with scCO_2_ at the start of the reaction; however, the solubility of the CTA must not be too great for scCO_2_ in order to avoid that the CTA stays in the continuous (scCO_2_) phase. It is important that the growing polymer chain with CTA is transferred to the disperse phase (out of the CO_2_ phase). In other words, a CTA must be soluble but not too soluble during the RAFT polymerization process. Four more CTAs were tested, all with one more or less CO_2_/polymer‐philic group (**Figure** **6**).

Only CPDB showed good control over the RAFT polymerization of MMA, this is due to being “averagely” soluble in scCO_2_, whereas PDMAT, CPAD, and CTPPA are more soluble in scCO_2_. PDMAT, CPAD, and CTPPA all have a carboxylic acid group which has good affinity toward scCO_2_. This suggest that these CTAs stay in the scCO_2_ phase and are thus less likely to partition in the growing polymer particles. Furthermore, PDMAT has a short alkene chain, which is not polymer‐philic, and thus the solubility is too high. CTPPA has an extra C≡N group, which makes it even more CO_2_‐philic. Finally, CPAD does have a long alkene group that is polymer‐philic. However, due to the extra C≡N group, it is still too soluble in scCO_2_ to properly control the polymerization.

**Figure 7:**
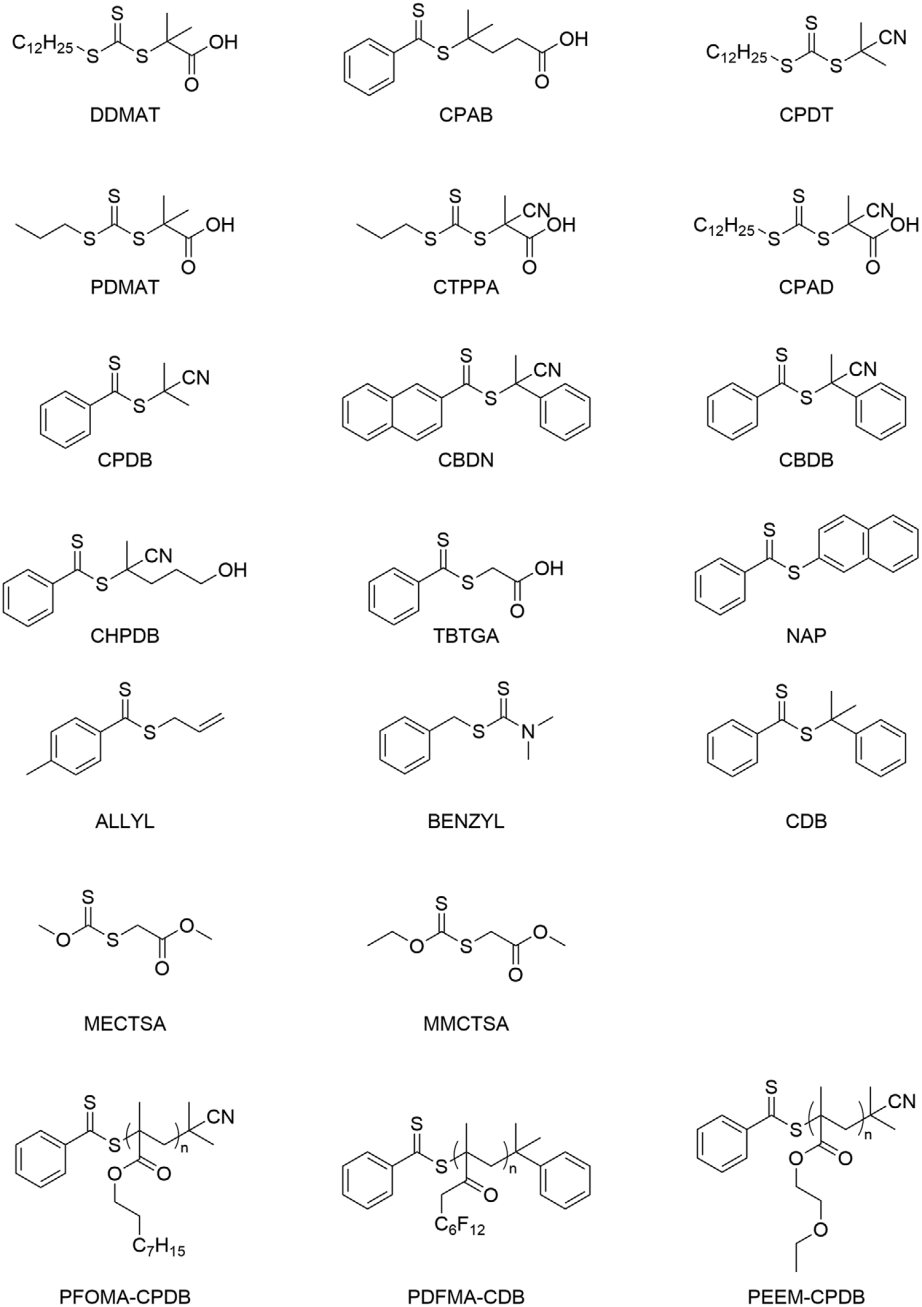
Chemical structures of CTAs used for RAFT polymerization in scCO_2_.

Another change to the RAFT polymerization is the use of macroCTAs instead of the traditional CTAs. These macroCTAs are best be described as CTA functionalized homopolymers. In some cases a stabilizer that can also act as a macroCTA is employed. The backbone is often a fluorinated polymer that can act as an in situ stabilizer. It must however be noted that the use of fluorinated polymers decreases the sustainability of polymerization in scCO_2_. It has been observed that the inhibition step (2) proceeds faster for macroCTAs. In the next few sections, an overview is given of all published RAFT homopolymerizations in supercritical CO_2_ with an overview in **Table** **1** and a general overview of the structures given in **Figure** **7**.

**Table 1:** RAFT homopolymerization reported in the literature for various monomers and CTAs.

Polymer	CTA	P [bar]	T [°C]	M_n_ [kDa]^a)^	Conversion [%]^a)^	Đ	References
PMMA	CBDN	276	65	28.7	99	1.2	[59]
	CBDB	276	65	26.2	98	1.1	[59]
	CPDB	276	65	24.6	94	1.1	[59]
	CHPDB	276	65	26	99	1.2	[59]
	PFOMA‐CPDB^b)^	276	65	76	99	1.2	[60]
	TBTGA	350	75	20	95	1.5	[61]
	CDB	300	80	153	78	1.8	[48]
	CPDB	276	65	70.5	99	1.2	[62]
	PDFMA‐CDB^b)^	300	70	108	99	1.4	[63]
PS	TBTGA	300	80	6.5	36	1.2	[64]
	NAP	300	80	2.7	18	1.6	[64]
	ALLYL	300	80	4.2	27	1.2	[64]
	BENZYL	300	80	14.7	33	2.7	[64]
	CDB	300	80	30	17	1.1	[47]
PVAc	MMCTSA	345	65	37	36	1.4	[65]
	MECTSA	345	65	49	37	1.6	[65]
PFHEMA	CPDB	330	60	54	40	1.2	[66]
PDMA	PEEM‐CPDB^b)^	300	60	22	54	1.38	[67]
P4AM	PEEM‐CPDB^b)^	300	60	37	64	1.38	[67]
PMBL	CPDB	300	80	20	93	1.5	[16]

^a)^ Highest reported conversion and molar mass;

^b)^ Starting from macroCTAs.

### 4.3 RAFT Polymerization of Methyl Methacrylate in Supercritical CO_2_

**Figure 8:**
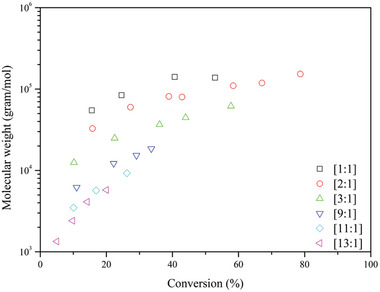
MMA polymerizations at different [RAFT]:[AIBN] ratios at 300 bar and 80 °C.

The most researched monomers for RAFT polymerization in scCO_2_ are styrene and methyl methacrylate. The first RAFT polymerization of MMA was performed in 2004 by Arita et al.^[^ ^48^ ^]^ In their study cumyl dithiobenzoate (CDB) was used as CTA. Different ratios of [CTA]:[AIBN] were investigated, starting from [1]:[1] till [13]:[1]. As expected, the highest conversion was obtained with the lowest concentration of CTA and the best control (low Đ) was obtained when having the highest concentration CTA, see **Figure** **8**.

Relatively good control over the polymerization was observed for a [RAFT:AIBN] ratio of 1:1.05 with a corresponding Đ ranging from 1.2 to 1.9. Increasing the [RAFT:AIBN] ratio resulted in a higher control with Đ values going as low as 1.05. However, conversions were lower and only reached 20% after 3 h with a [RAFT]:[AIBN] ratio of [13]:[1]. Retardation of the polymerization is dependent on the concentration of the CTA, meaning that a higher CTA concentration decreases the polymerization rate. Furthermore, an increase in retardation is observed when scCO_2_ is the medium instead of toluene. From all experiments a pseudo first order reaction of the polymerization can be observed due to a consistent linear increase of molar mass by conversion. It is noteworthy that at higher AIBN concentrations the theoretical molar mass by Equation (2) is more suited, while for low AIBN concentration the molar mass of the final polymer is more in agreement with Equation (1).

Howdle studied the RAFT dispersion polymerization of MMA in supercritical CO_2_.^[^ ^62^ ^]^ A stabilizer, PDMS‐MA, was added to the system that has shown to be advantageous for dispersion polymerization. Without the CTA, control over the polymerization process is poor and a polymer with an M_n_ of 73 kDa with a high Đ of 2.59 was obtained. In addition, a kinetic study was performed to monitor the molar mass increase as a function of the conversion.

The polymerization rate is reduced considerably in the presence of a RAFT agent, evident from a significant decrease in the monomer conversion from 99% to 17% after 10 h. This was attributed to lower inhibition rates in supercritical CO_2_ caused by the retardation effect of the CTA. High conversions and a polymer with a low Đ are obtained after 14.5 h. The conversion is linearly proportional to the molar mass, suggesting that chains grow uniformly. Using a [1]:[1] RAFT to AIBN ratio led to great control over the system with Đ’s lower than 1.2 and M_n_≈ Mn_th_.

To further verify the chain end functionality, of the synthesized polymers with molar masses of 23 and 29 kDa underwent chain extension by reintroducing them with MMA and styrene, along with additional AIBN. Namely, these polymers are terminated with the CTA groups (macroCTA), thus introducing the possibility of reinitiating the polymer chain. In general, good control is observed by the addition of fresh monomer, with no extra nucleation, meaning the new monomer is reacting with the already formed polymer (macroCTA). To remove the end groups of the CTA in the polymer, excess AIBN was introduced, resulting in a colorless and odorless final product.

Howdle et al. also performed an extensive study on the RAFT of MMA in supercritical CO_2_, in which four different di‐thioesters were investigated as CTAs, namely CBDN, CBDB, CPDB and CHPDB.^[^ ^59^ ^]^

**Figure 9:**
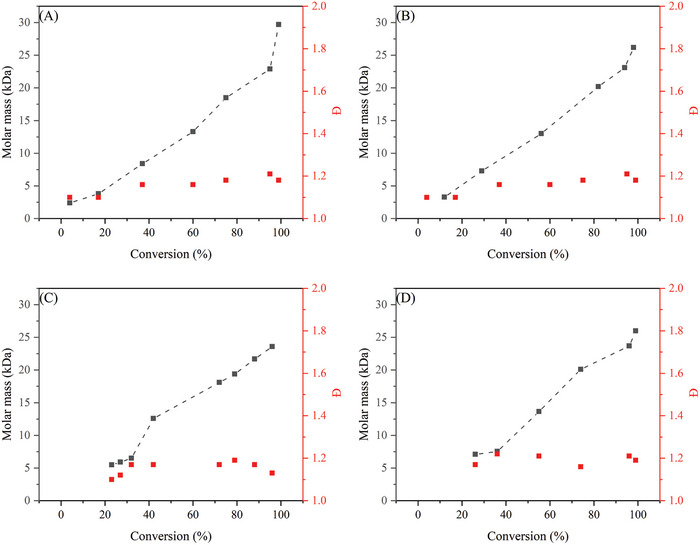
RAFT polymerization results of MMA in scCO_2_ with different CTAs; A) CBDN, B) CBDC, C) CPDB and D) CHPDB.

Two studies were performed on the RAFT polymerization of MMA: i) the influence of the CTA on the control of the polymerization and ii) the influence of the stabilizer concentration on the morphology of the formed particles. After ≈24 h full conversion was reached for each individual CTA. Great control over the system was shown with low Đ’s ranging from 1.1 to 1.22. All RAFT polymerizations showed pseudo first order reaction kinetics as can be seen in **Figure** **9** due to a linear correlation between molar mass and conversion.

However, long inhibition periods were observed in the tested CTAs. From a molecular structure point of view, it was shown by Rizzardo et al. that having a bulkier leaving group (R) is more beneficial for electron‐donating groups.^[^ ^68^ ^]^ A di‐thioester has an advantage over a tri‐thioester, because the higher electron withdrawing effect of the Z group provides enhanced addition to the C═S double bond for MMA RAFT‐polymerization. Also, all CTAs have a CO_2_‐philic group (C≡N) and a polymer‐philic group (benzene), adhering to the soluble but not too soluble criterion in supercritical CO_2_. However, CTA CBDN and CBDB experienced a long induction period due to the bulky R group, as was also investigated by Rizzardo. The stabilizer PDMS‐MA was added into the system to ensure the formation of spherical polymer particles inside the dispersed medium. By adding a small amount (2.5 wt.%), a significant increase in conversion and a decrease in dispersity was seen.

**Figure 10:**
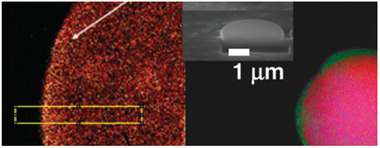
Polymer self‐assembly process taken from Zong and adjusted (carbon (red), oxygen (pink) and green (fluorine)).^[^ ^60^ ^]^

To reduce the long inhibition period for the CTAs in supercritical CO_2_, macroCTAs were being tested in the work of Zong et al.^[^ ^60^ ^]^ A fluorinated CTA, terminated with 2‐cyano‐2‐propyl benzodithioate, was used. The backbone of this macroCTA is a fluorinated polymer that can also act as an in situ stabilizer of the system, PFOMA. By using this macroCTA, the inhibition period decreased from ≈10 to ≈1 h. Control of the system was kept constant with full conversion reached after 20 h, exhibiting pseudo‐first order kinetics. SEM images show excellent spherical morphology at any stage of the reaction path. When observing elemental density of the formed particles, a higher concentration of fluorine on the outside of the particle is observed. The CO_2_‐philic chains, in this case the PFOMA block, of the polymer migrated to the outside, during the self‐assembly process of the polymer, as shown in **Figure** **10**.

Vivaldo–Lima et al. investigated the influence of pressure, temperature and stabilizer concentration on the polymerization of MMA, with TBTGA as CTA and Krytox 257 FSL as stabilizers.^[^ ^61^ ^]^ High monomer conversions were reached after 20 h and the polymerization proceeds in a relatively controlled manner. Polymerizations at higher pressure and temperature resulted into higher conversions and better control. By increasing the pressure, a higher density of the supercritical solvent is obtained. This correlates to enhanced solvent power, so that the growing polymer phase will be longer in the undispersed phase. Higher temperature had a positive influence on the individual rate constants of the reactions involved in the RAFT polymerization scheme.

Xu et al. polymerized a macroCTA agent of poly(dodecafluoroheptyl methacrylate) with MMA in a two step polymerization method.^[^ ^63^ ^]^ First, a macroCTA was synthesized using DFMA and CDB at different molecular masses. Three different macroCTAs were being used to polymerize 12 different block copolymers as shown in **Table** **2**.

**Table 2:** MacroCTA synthesized by Xu et al.

macroCTA	Molecular mass [kDa]	Đ
PDFMA_15_‐CDB	3.7	1.07
PDFMA_32_‐CDB	10.9	1.11
PDFMA_55_‐CDB	17.6	1.12

**Figure 11:**
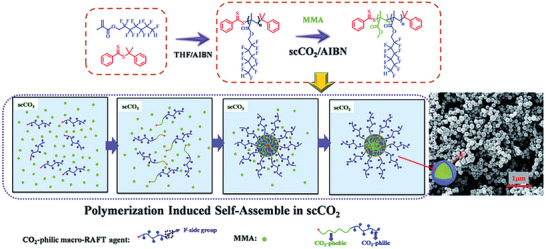
Synthesis of fluorinated nanoparticles via RAFT dispersion polymerization‐induced self‐assembly using fluorinated macro‐RAFT agents in supercritical carbon dioxide.

Next the second monomer, MMA, was added with an additional initiator to block copolymerize. By using a macroCTA that is highly fluorinated, no stabilizer was needed for the reaction in scCO_2_. Furthermore, the growing polymer underwent a polymerization‐induced self‐assembly. This resulted in the formation of spherical particles, where the fluorinated block migrated to the outside due to its CO_2_‐philicy, encapturing the more CO_2_‐phobic MMA block. The same observation was found for the use of a fluorinated Macro‐RAFT agent by Zong et al.^[^ ^60^ ^]^ A schematic drawing of this self‐assembly is presented in **Figure** **11**.

Different block copolymers were polymerized with lengths ranging from 16 to ≈100 kDa. High conversions were obtained ranging between 96% and 100%. Dispersion of the polymerization takes place when a critical MMA chain length has been formed. The effect on the length of the MMA block was studied by keeping the fluorinated macroCTA fixed. With an increasing degree of polymerization of the MMA and thus a larger PMMA block, the formed particles will be more spherical. When the CO_2_‐philic PMMA chain surpasses a critical chain length a microphase segragation occurs to form spherical aggregates with a PMMA core that are stabilized by the PDMA outer shell. When having a higher starting chain length of the PDFMA block, the particle size decreased and retained a more narrow particle size distribution. Having a higher CO_2_ pressure results in a higher solvancy of the polymer. When increasing the reaction pressure, the residence time of the growing polymer chain is longer in the homogenous phase (scCO_2_), thus increasing the average particle size. Furthermore, the particle size distribution decreased from 1.61 to 1.06 when the reaction pressure was increased from 100 to 300 bar.

### 4.4 RAFT Polymerization of Styrene in Supercritical CO_2_

**Figure 12:**
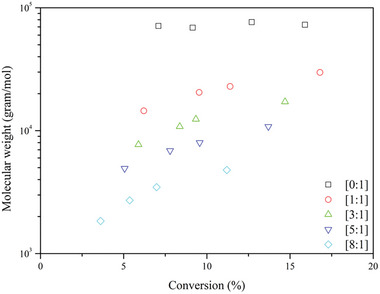
Styrene polymerizations at different [RAFT]:[AIBN] ratio's at 300 bar and 80 °C.

Next to MMA, styrene is typically used for RAFT polymerization in scCO_2_. Usually, styrene is used for block copolymerization.^[^ ^69^ ^]^ The first reported RAFT polymerization of styrene in supercritical CO_2_ was by Arita.^[^ ^47^ ^]^ RAFT polymerization reactions were carried out for short periods to determine the conversion and dispersity for styrene in supercritical CO_2_, and the results are given in **Figure** **12**. The effect of varying RAFT concentrations was measured, and the difference between RAFT polymerization in scCO_2_ and toluene were also investigated. When no CTA was added to the system, mediocre control in the early phase was observed. Furthermore, higher molar masses with a high Đ are obtained compared to experiments when a CTA is added to the system, thus contributing to less control over the polymerization process. It was also evident that higher RAFT concentrations led to lower reaction rates. When the [RAFT]:[AIBN] ratio is increased above [3]:[1] no significant improvement in the control over the system was observed. Higher concentrations of CTA did result in lower molar masses as well as lower conversion and slower polymerization kinetics. However, no polymerization experiments were carried out to reach higher conversions, which would give a better understanding on the controllability of the system.

**Figure 13:**
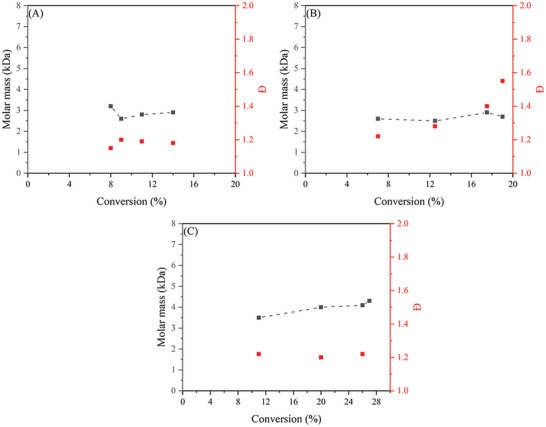
RAFT polymerization results of styrene in scCO_2_ with different CTAs; A) TBTGA, B) NAP, and C) ALLYL.^[^ ^64^ ^]^

Lima et al. conducted a study on the impact of various CTAs and stabilizer concentrations on the RAFT polymerization of styrene in supercritical CO₂.^[^ ^64^ ^]^ In addition, a study on the [RAFT]:[AIBN] was performed on the most suitable CTA of the study. The four different RAFT controllers being investigated were TBTGA, NAP, ALLYL, and BENZYL. For the first three CTAs, reasonable to good control is given over the polymerization, see **Figure** **13**. For the final CTA (BENZYL), poor control with high conversion was seen. The CTA that gave the best control over the system, however, gave the lowest conversion over time, ≈13% after 24 h.

With TBTGA being the best performing CTA in terms of polymer dispersity, a study on the different stabilizer concentrations was conducted. Five different wt.% concentrations of the starting monomer have been used with similar results for the majority of stabilizer wt.% (3–10%). Increasing the concentration of the stabilizer does not affect the polymer dispersity of the formed polymer, as it was remained constant at ≈1.15. There appeared to be no noticeable benefits of increasing stabilizer concentration above 3 wt.% on the monomer conversion. It must be noted, however, that these reactions were only carried out for short reaction periods by the authors. To get a better understanding of the behavior of the stabilizer it is suggested to extend this polymerization to higher conversions, i.e., longer reaction times. This is due to lower solubility of the longer polystyrene chains when the conversion increases. Finally, the effect of higher RAFT/AIBN ratios was studied. With increasing concentration of the CTA the conversion dropped significantly with the highest conversion being 36% at [RAFT]:[AIBN] being 0.5 and the lowest conversion being 14% at [RAFT]:[AIBN] of 2. A small decrease in polymer dispersity was observed when increasing the RAFT concentration. However, this is negligible due to the overall decrease in conversion. The results from this experiment suggest that addition and fragmentation reactions occurred in the dispersed phase as hypothesized and this in agreement with other published work.

### 4.5 RAFT Polymerization of Vinyl Acetate in Supercritical CO_2_

**Figure 14:**
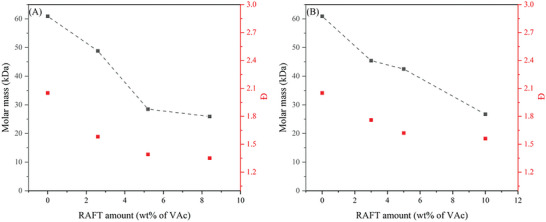
RAFT polymerization results of VAc in scCO_2_ with different CTAs after 10 h by Pham et al; A) MMCTSA, B) MECTSA.^[^ ^65^ ^]^

In the previous sections a stabilizer was needed for successful RAFT polymerization in supercritical CO_2_. Pham et al. decided to perform the RAFT polymerization of vinyl acetate (VAc), which is a highly soluble polymer in scCO_2_.^[^ ^65^ ^]^ Those formed polymers were poly(vinyl acetate)‐based that were functionalized with xanthates end groups. Two different xanthates were used during the experiments: methyl(ethoxycarbonothioyl) sulfanyl acetate (A) and methyl(methoxycarbonothioyl) sulfanyl acetate (B), see **Figure** **14**. A study on the RAFT:AIBN ratio was performed for 10 h, measuring Đ and molar mass. The AIBN concentration was kept constant during each experiment while the RAFT concentration increased. It is evident that the addition of the macroCTA improves the uniformly chain growth of the polymerization greatly, lowering the Đ from 2.05 to 1.35 for PVAc‐X1 at 25.9 kDa. Better control over the reactions is observed when the macroCTA concentration was increased. With the small amount of CTA, Equation 2 provides a better estimation of the final molar mass. The formed macroCTA was used for chain extension experiments by adding vinyl pivalate (VPi) to synthesize a PVAc‐*b*‐PVPi block copolymer.

### 4.6 RAFT Polymerization of 2‐(Perfluorohexyl)ethyl Methacrylate (FHEMA) in Supercritical CO_2_

Chekurov et al. researched the RAFT polymerization of 2‐(perfluorohexyl)ethyl Methacrylate in supercritical CO_2_ as well as in trifluorotoluene (TFT).65 For both reaction mediums the commercially available CTA CPDB was used. The main research question in this study was whether supercritical CO_2_ can be replaced by the organic solvent. It was concluded that the RAFT polymerization of FHEMA in scCO_2_ proceeded under homogenous conditions with reasonably low dispersity. Due to the good solubility of the fluorine‐based monomer, no stabilizer was needed in this process. The molar mass characteristics of the polymers obtained in scCO_2_ and TFT showed that the CPTB efficiency is higher in scCO_2_, meaning that RAFT polymerization of fluorine‐based acrylates is a better alternative compared to the conventional solvents.

### 4.7 RAFT Polymerization of 2‐(Perfluorohexyl)ethyl Methacrylate (FHEMA) in Supercritical CO_2_

**Figure 15:**
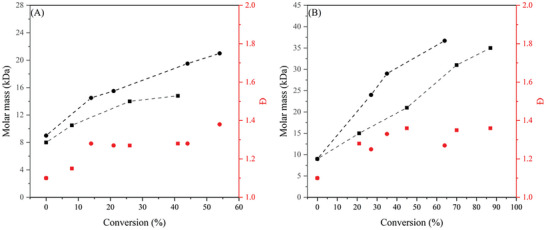
RAFT polymerization results of A) DMA and B) 4AM in scCO_2_ with macroCTA by Hawkins. Squares and circles have a [Monomer]_0_/[MacroCTA] = 200 and 400.

A first report on the RAFT polymerization of an acrylamide in scCO_2_ has been reported in 2017. Hawkins et al. synthesised a macroCTA from 2‐ethoxyethyl methacrylate, terminated with the CTA 2‐cyano‐2‐propyl benzodithioate.^[^ ^67^ ^]^ Two different acrylamides were investigated for the copolymerization, N,N‐dimethylacrylamide (DMA) and 4‐acryloylmorpholine (4AM). Both macroCTA and formed block polymers were deemed to be insoluble in scCO_2_ at 65 °C and 300 bar, making it a heterogenous polymerization that dispersed instantly. The polymerization was also done in solution to find out the differences between the reaction medium. Polymerization rates were higher in solution than in CO_2_, which can be attributed to a few phenomena. First, the initiator decomposition rate is lower and the termination rate is higher in scCO_2_ compared to organic solutions. Second, due to the poor solubility of the forming polymer/macroraft and the good solubility of the second monomer the monomer concentration at the main locus of the polymer is lower, slowing down the polymerization. The molar mass seems to grow linearly with the conversions, which suggest that there is good control over the system with reasonable obtained dispersities ranging from 1.1 to 1.35, indicated in **Figure** **15**.

### 4.8 RAFT Polymerization of *α*‐Methylene‐*γ*‐Butyrolactone in Supercritical CO_2_

**Figure 16:**
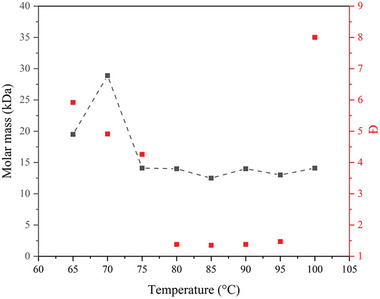
Results of RAFT polymerization of *α*‐MBL in scCO_2_ after 24 h at different temperatures.

Versteeg et al. performed RAFT polymerization of *α*‐methylene‐*γ*‐butyrolactone (*α*‐MBL) in supercritical CO_2_.^[^ ^16^ ^]^ *α*‐MBL is an interesting compound because it is not only bio‐renewable but its polymerization leads to PMBL, which has a high glass transition temperature making it attractive for high temperature applications. The study was carried out over the temperature range of 65–105 °C. When no CTA was added polymers with a high Đ were obtained, while CPDB was used as the CTA in other experiments. The polymerizations between 65 and 75 °C and 95+ °C showed limited control and closely follows the free radical polymerization. Overall, good control over the polymerization was observed for temperatures between 80 and 95 °C as can be seen in **Figure** **16**.

However, there was still monomer present in the final product after venting off the CO_2_. Due to the high *T_g_* polymer residual monomers gets entrapped within the polymer matrix as the polymer chains do not have any mobility; this will ultimately result in the need for an extra clean up step.

## 5 RAFT Dispersion Block Copolymerization in Supercritical CO_2_

In the previous section, the homopolymerization of different monomers is discussed. An argument can be made that block copolymers are synthesized as well. However, this always included a two‐step synthesis process where soluble macroCTAs were synthesized in solution or bulk and thus not in supercritical CO_2_. For that reason, we refrain to call these block copolymerizations in supercritical CO_2_. In the following section, the state of the art RAFT block copolymerization is discussed in which both blocks are synthesized in scCO_2_. To the best of our knowledge, there have been only three published articles in which the block copolymer was fully synthesized in scCO_2_ under the group of Howdle et al.^[^ ^70^, ^71^, ^72^ ^]^

**Figure 17:**
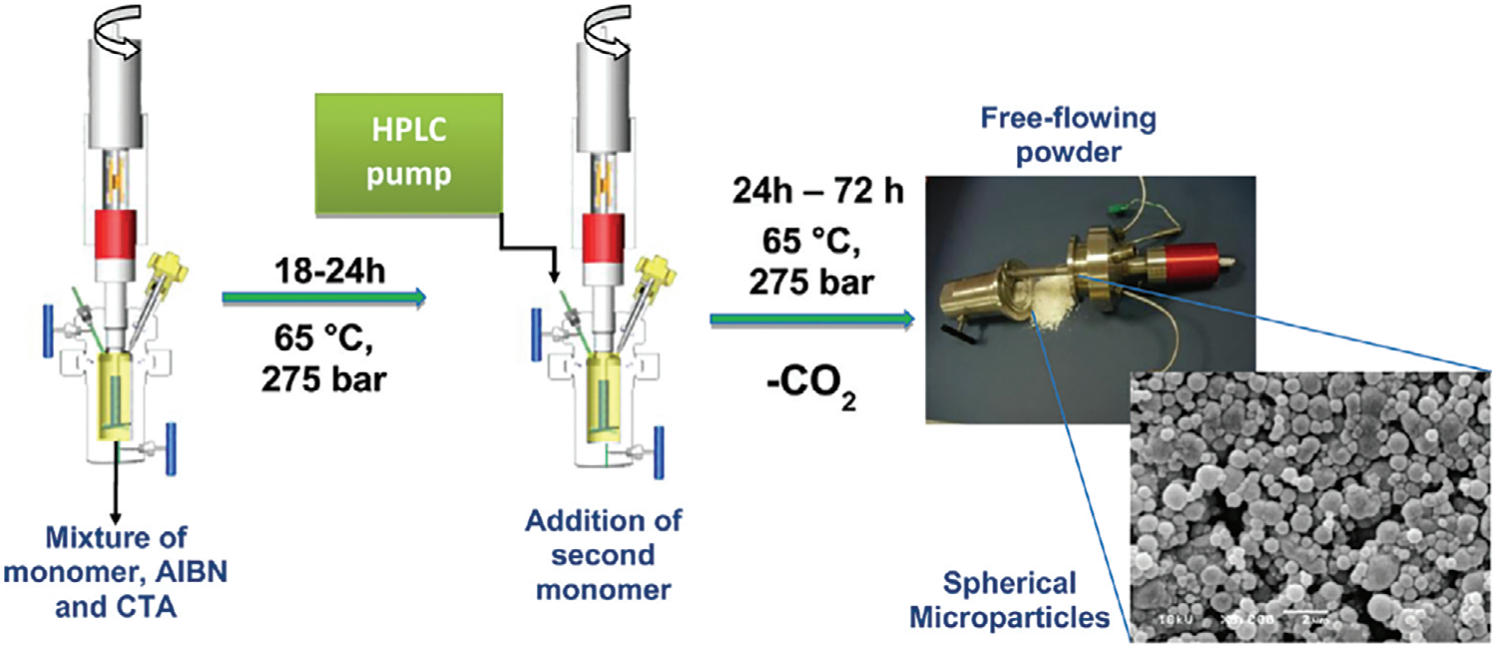
Experimental one‐pot synthesis set‐up used by the Howdle group. Reprinted (adapted) with permission from Jennings et al. Copyright {2012} American Chemical Society.

The first one‐pot synthesis of RAFT block copolymerization was reported by Jennings et al.^[^ ^70^ ^]^ Five different block copolymers were made consisting of a PMMA precursor followed by a P4VP/PS/PBzMA/PDMAEMA/PDMA block. In **Figure** **17** a schematical set up is provided in which the one‐pot synthesis can be seen. In the first step a macroCTA is made from MMA and a trithiocarbonate (DATC). This CTA has been shown to exert good control over the polymerization of vinyl monomers.^[^ ^73^ ^]^ Second, four different monomers are added under pressure to form diblock copolymers.

The block copolymer synthesis exhibited, in general, good molar mass control and low dispersity for methacrylate copolymers as shown in **Table** **3**. Furthermore, conversions typically reached 90+% for all polymerizations. However, for styrenics and acrylamides low dispersity is not observed because these monomers have a higher tendency to terminate by combination. The low viscosity and high diffusivity of scCO_2_ facilitate the efficient plasticization of PMMA. This leads to excellent access for the growing polymer chain ends and the RAFT agent to incoming monomers, ensuring that the polymerization process remains within the microparticle and promotes effective block copolymer formation. Additionally, the reversible chain transfer mechanism ensures that the RAFT agent remains attached to a polymer chain throughout the reaction, keeping it within the particle.

**Table 3:** Results of block copolymerization by Jennings et al.^[^ ^71^ ^]^

Polymer	M_n,theo_ [kDa]	M_n,exp_ [kDa]	Đ	Pressure [bar]	Temperature [°C]
PMMA(45)‐*b*‐P4VP(15)	60	69	1.99	275	65
PMMA(30)‐*b*‐P4VP(30)	60	68	1.98	275	65
PMMA(15)‐*b*‐P4VP(45)	60	61	1.71	275	65
PMMA(30)‐*b*‐PS(30)	60	57	1.68	275	65
PMMA(75)‐*b*‐PBzMA(25)	100	83	1.32	275	65
PMMA(50)‐*b*‐PBzMA(50)	100	89	1.32	275	65
PMMA(45)‐*b*‐PDMAEMA(15)	60	48^a)^	1.24	275	65
PMMA(30)‐*b*‐PDMAEMA(30)	60	44^a)^	1.33	275	65
PMMA(75)‐*b*‐PDMA (25)	100	94	1.58	275	65
PMMA(50)‐*b*‐PDMA(50)	100	94	1.69	275	65

^a)^ Due to the difference in hydrodynamic volume, the molar masses from GPC are lower.

**Figure 18:**
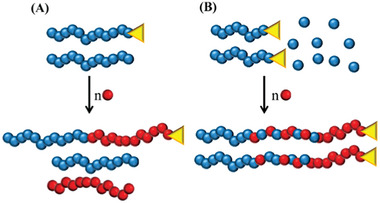
Decrease of blocking efficiency due to A) formation of dead polymer chains or B) formation of tapered blocks due to unreacted monomer.

In the follow up study, Jennings al. presented an one‐pot synthesis for well‐defined polymeric particles via RAFT polymerization in supercritical CO_2_.^[^ ^71^ ^]^ It was previously established that block copolymers can be made with high conversion and control. However, a possible significant drawback of a block copolymerization in a one‐pot set‐up is that the purity of the block copolymer product can be compromised by loss of blocking efficiency due to dead chains (**Figure** **18**A) or formation of tapered blocks (Figure 18B).

**Figure 19:**
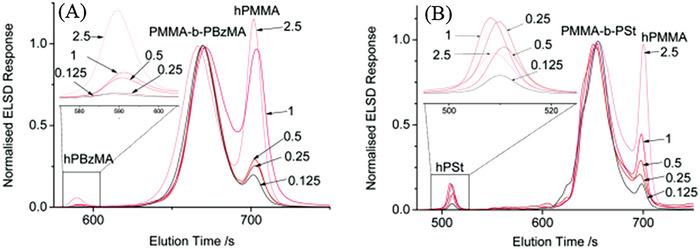
GPC curves of the block copolymerization of A) PMMA*‐b‐*PBzMA and B) *PMMA‐b‐PS* with different AIBN ratios.

Here, two types of block copolymers of different lengths were successfully synthesized with good control over the system. First, a homo‐polymer of PMMA was made inside the reactor and after 24 h additional monomer (in this study BzMA or styrene, respectively) and AIBN was added through an HPLC pump. Different ratios of AIBN were used to find the optimum reaction conditions. When the initiator concentrations were decreased, not only did the polymer dispersity drop but the blocking efficiency increased as well. This is explained as at lower AIBN concentrations the termination reactions are decreased. GPC analysis of the final polymer shows three distinctive peaks, which are homo‐polymer PMMA, homo‐polymer PBzMA and block copolymer PMMA‐b‐PBzMA. These impurities can arise from loss of chain end functionality resulting in dead polymer chains or unreacted monomer in the first step, resulting in a tapered block, see **Figure** **19**.

Blocking efficiency was determined via three different methods: GPC Deconvolution, GPEG, and GPC Dual detection. For all three methods, blocking efficiency increased by lowering the initiator concentrations. By applying the method of GPC deconvolution, three peaks were distinguished; namely, homo‐polymer PMMA, homo‐ polymer PBzMA and block copolymer PMMA‐b‐PBzMA for the block polymerization with BzMA. For block polymerization with styrene the peaks were homo‐polymer PMMA, homo‐polymer STY and block copolymer PMMA‐b‐PSTY. The height of the hPPMA peak decreases for both blockpolymerization with an increasing concentration of [AIBN] (2.5>1>0.5>0.25>0.125).

Alauhdin et al. used a high pressure X‐Ray Scattering (SAXS) to study the internal morphology development and evolution of a series of RAFT polymerized PMMA‐PS block copolymer microparticles. In contratry to the previous study a two pot synthesis was used so that there are two distinct pressurization/polymerization/depressurisation stages. This was employed because by adding additional stabilizer PDMS‐MA in the second pot the chain end functionality was improved. For block compolymerizaiton styrene was the chosen chemical as the formation of block copolymers in scCO_2_ can lead to different morphologies during the self‐assembly process, **Table** **4**. Examples of different morphologies being lamella, cylinders, gyriods and spheres, in which each one can influence the physical properties of the polymer. By using dispersion raft polymerization in scCO_2_ there is no need for an additional step to form these morphologies as self‐assembly happens during the synthesis. Due to the plasticising effect of the scCO_2_ medium, self‐assembly occurs at moderate temperatures, and blocking efficiency in scCO_2_ is as good as in conventional solvents.

**Table 4:** Block copolymerization results and morphology by Alauhdin et al.

Polymer	Mnt	Mnexp	Conversion	Đ	Morhoplogy
PMMA(31)‐PS(25)	70	56	79	1.5	Cylinder
PMMA(25)‐PS(50)	100	75	80	1.84	Cylinder/lamellae
PMMA(58)‐PS(12)	80	70.2	76	1.51	NA
PMMA(54)‐PMMA(44)	100	95.4	97	1.45	NA

For the monitoring of the morphology development a time‐resolved SAXS profile was recorded during chain extension of the PMMA chain. For the PMMA(31)‐PS(25) hexagonal cylinders were observed across the reaction. The PMMA(25)‐PS(50) polymer showed the same scattering of microparticulate withouth internal structure. At early reaction stages, spehrical PS domains are forming within the PMMA microparticles. With increasing PS block, a morphology change begins to form. The final product, being a mix of morphologies, consist of lamellar being predominant at the particle exterior and a hexagonal morphology being dominant at the inner exterior. For both the PMMA(58)‐PS(12) and the PMMA(54)‐PMMA(44) polymer the obtained SAXS profiles are initially featureless. The lack of order is attributed to its high degree of block asymmetry, positioning it toward the edges of the phase diagram where the formation of ordered structures becomes thermodynamically unfavorable.

## 6 Conclusion

In this review the state of the art of RAFT induced homopolymerization of different monomers with corresponding chain transfer agents in scCO_2_ as solvent has been investigated. A characteristic for the RAFT induced polymerization is the controlled radical polymerization, also called reversible deactivation radical polymerization. As the use of VOCs will be strictly limited or even banned in future processes, the use of scCO_2_ is a significant environmentally attractive alternative compared to the use of VOCs in conventional RAFT polymerization. Furthermore, the selection of the most optimal CTA is of high importance for the success of the RAFT polymerization as it affects the both the Đ and the molar mass. Also the presence and the type of stabilizers are inevitable to control the product specifications with the exception of fluor‐based polymers. The presence of these stabilizers, however, need to be removed and recycled, otherwise the circular or sustainability of the RAFT induced polymers cannot be completely regarded as “green products”. Unfortunately, it is not possible to produce polymers with a high molar mass via RAFT induced polymerization in scCO_2_ without the use of fluorinated compounds. At a critical length, depending on the type of polymer, the maximum solubility of the polymer is attained and precipitation of the polymer in the scCO_2_ will start. Overall, it can be concluded that the RAFT induced polymerization in scCO_2_ is a promising technology to arrive at a circular, sustainable production route for low molar mass polymers.

## Conflict of Interest

The authors declare no conflict of interest.
